# Adsorption of Sodium Dodecyl Sulfate on Ge Substrate: The Effect of a Low-Polarity Solvent

**DOI:** 10.3390/ijms13077980

**Published:** 2012-06-28

**Authors:** Rommel B. Viana, Albérico B. F. da Silva, André S. Pimentel

**Affiliations:** 1 Department of Chemistry and Molecular Physics, Institute of Chemistry of São Carlos, University of São Paulo,13560-970, São Carlos, SP, Brazil; E-Mail: alberico@iqsc.usp.br; 2 Department of Chemistry, Pontifical Catholic University of Rio de Janeiro, 22453-900, Rio de Janeiro, RJ, Brazil; E-Mail: pimentel@qui.puc-rio.br

**Keywords:** anionic surfactant, vibrational spectra, dichroism, deposition

## Abstract

This paper describes the adsorption of sodium dodecyl sulfate (SDS) molecules in a low polar solvent on Ge substrate by using Fourier transform infrared-attenuated total reflection (FTIR-ATR) spectroscopy and atomic force microscopy (AFM). The maximum SDS amount adsorbed is (5.0 ± 0.3) × 10^14^ molecules cm^−2^ in CHCl_3_, while with the use of CCl_4_ as subphase the ability of SDS adsorbed is 48% lower. AFM images show that depositions are highly disordered over the interface, and it was possible to establish that the size of the SDS deposition is around 30–40 nm over the Ge surface. A complete description of the infrared spectroscopic bands for the head and tail groups in the SDS molecule is also provided.

## 1. Introduction

Surfactant deposition on substrates has been widely studied due to its significance in both applied and fundamental processes [1]. The determination of the packing, and ordering, and their relation to the properties of the surfactant aggregates is of fundamental importance [2,3]. These properties have been widely investigated and used to infer aggregation on substrates. However, the understanding of the structure and conformation of these aggregates has been limited due to a lack of suitable tools to determine the ordering and packing of the surfactants on substrates [4–7]. Two important tools to investigate the interaction between surfactant and substrate are Fourier transform infrared-attenuated total reflection (FTIR-ATR) spectroscopy and atomic force microscopy (AFM). While AFM has been providing superficial geometric considerations of the bulk assembly of surfactants and specific surfactant-surface interactions [8–14], FTIR-ATR can afford an excellent insight into the structure and conformation of these molecular systems [3,15]. In the past, the inability of infrared spectroscopists to assign some band features has been the main barrier to advance in studying the organization and conformation of few surfactants monolayers using FTIR-ATR [4–7,16].

Recently, the use of solvents with low dielectric constant has been of concern in the advance of nanotechnology [17–19]. Chlorofom (CHCl_3_) solvent has been recently employed in the development of metal oxide nanorods [17,18] and nanowires [19], and, in addition, surfactants have also assisted in the growth process of different nanorods [20,21]. It is important to mention that the influence of the medium dielectric constant on the surfactant adsorption process has been receiving almost no attention throughout the years [22]. In addition, germanium substrates are better semiconductors than silicon ones and, as described by Kandel and Kaxiras [23], surfactants have a high impact in the development of semiconductor thin-films. Germanium substrates demonstrate relevance in the field of III–V epitaxy [24,25], in the manufacture of solar cells [26,27] and also in the development of nanowires [28,29] and until now there are few studies concerning the adsorption of surfactants on a germanium substrate [30]. Therefore, the purpose of the present study is to extend the range of reliable infrared measurements of adsorption of anionic surfactants on Ge substrate by using the FTIR-ATR and AFM techniques. The different approach of this work is the application of CHCl_3_ as the bulk solvent, which presents a dielectric constant 16 times smaller than the water value. Special attention is devoted to the CH_2_ asymmetric stretching and scissoring bands, and also SO_4_^=^ asymmetric features, to observe the organization degree of the surfactant molecules adsorbed on the Ge substrate.

## 2. Results and Discussion

### 2.1. SDS Deposition and Adsorption Analysis

The surfactant deposition time was optimized by varying it from a few minutes to 2 h. Li and Tripp [13] observed that CTAB takes from 50 to 100 min to deposit on TiO_2_, while SDS requires around 50 min to adsorb on the CTAB over TiO_2_. In our investigation it was observed that the amount of surfactant molecules deposited on Ge substrate does not change substantially after depositing them for more than 10 min. Then, our choice was to deposit the surfactant for 20–40 min. This deposition time range was used by Biswas and Chattoraj [31], who showed that deposition reached at least 50% within 10 min. The authors also demonstrated that there is an enhancement of the deposition with increasing temperature [32].

It was observed that the transfer ratio is around a few percent or less. The transfer ratio decreases as the number of SDS molecules increases on the CHCl_3_ subphase. Unfortunately, the SDS deposition forms aggregates when the transfer ratio is around 3.5%. At this transfer ratio, the surface coverage is not complete, while transfer ratios smaller than 0.1% lead to a complete coverage of the Ge substrate. Besides, the transfer ratio of these molecules to the Ge substrate is known to be close to one using ultra pure water as a subphase [33]. It is important to mention that the surface tension of an apolar organic solvent is usually much smaller than that for water, which makes it impossible to completely transfer the surfactant to the Ge substrate. Esumi *et al.* [34] also suggested that a second layer of deposited surfactant was absent in solvents with low dielectric constants. Few tests were also performed with CCl_4_, however, CHCl_3_ was found to be a better choice than CCl_4_ as subphase. The SDS deposition using CHCl_3_ as a subphase was around 48% larger than that using CCl_4_. This different adsorption values between CHCl_3_ and CCl_4_ solvents is due to the different dielectric constants. The small dielectric constant of CCl_4_ may lead to the formation of large aggregates [35], which may increase the concentration in the subphase and reduce the adsorption on Ge substrate. It is important to mention that CCl_4_ is highly toxic and also considered carcinogenic, while CHCl_3_ demonstrated a lower toxicity.

**Figure 1:**
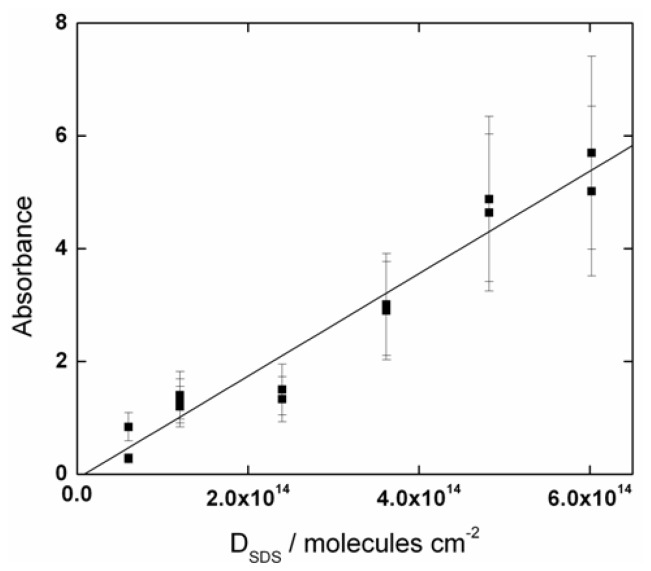
The infrared absorbance of the CH_2_ asymmetric stretching band for sodium dodecyl sulfate (SDS) molecules on Ge substrate. The density of SDS molecules (D_SDS_) was estimated using the casting technique assuming complete substrate coverage.

The number of SDS molecules, N_SDS_, on the Ge substrate was obtained by using the casting technique [11,36,37] under the assumption of homogeneous deposition and that the SDS molecules remain after deposition. Because this technique is well known to produce disordered molecules over the substrate [11], it is suitable for the purpose of this investigation. The procedure was to cast increasing amounts of SDS molecules, and to evaluate the absorbance of the CH_2_ asymmetric stretching band. From three SDS/water stock solutions (1, 20, and 500 mM), the number of SDS molecules was varied from 0.3 to 3.0 × 10^15^ molecules on the Ge substrate. As the surface area of the Ge element is 5 cm^2^, the density of SDS molecules on it varied from 0.6 to 6 × 10^14^ molecules cm^−2^. The solution volume added on the Ge substrate was varied from 2 to 50 μL. At low solution volumes (<10 μL), it was difficult to cover the whole substrate before evaporating. In these cases, the assumption that the SDS molecules completely covered the Ge substrate was used. Then, the Ge substrate was kept in a desiccator coupled to a vacuum pump for 10 min to eliminate the excess water. However, this step was not necessary when small aliquots (2 to 8 μL) were used, due to fast water evaporation at room temperature. Two critical parameters are considered: Aliquot volume and solution concentration. When volumes larger than 20 μL or concentrations higher than 100 mM are used, the reproducibility for the absorbance of the CH_2_ asymmetric stretching band was significantly reduced to 15%. The amount of SDS molecules deposited on the Ge substrate allows to one estimate its density on the Ge element as presented in Figure 1, which shows a straight line with a correlation coefficient of 0.97. Then, the density was estimated using the assumption that the SDS depositions cover 90% of the Ge substrate.

### 2.2. Atomic Force Microscopy Results

**Figure 2:**
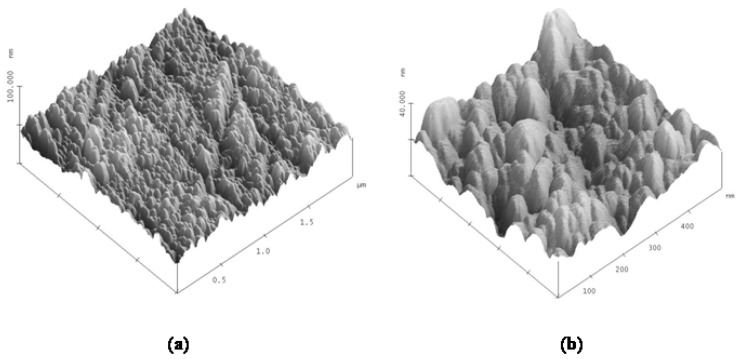
The atomic force microscopy (AFM) images of the SDS molecules deposited on Ge substrate using the technique are presented at (**a**) 2 × 2 μm^2^ and (**b**) 500 × 500 nm^2^ resolutions. The *z*-axis refers to the depth used to estimate the thickness.

The AFM images with 2 × 2 μm and 500 × 500 nm resolution are shown in Figure 2. It is important to point out that AFM results do represent directly a definite degree of conformational ordering of the SDS molecules deposited for the superficial layer. As can be seen, SDS molecules are highly disordered, at least at the air interface, indicating that the assumption applied for *all-trans* CH_2_ configuration must be carefully considered. It is very well known that SDS deposits as hemicylinders on graphite and mica substrates [8–10]. However, the AFM images in Figure 2a also indicate that the surfactant deposition may be overlapped and entwined like bat structures on the Ge substrate. The size of these bat structures is around 30–40 nm, which is 5 to 7 times larger than that for hemicylinders, ~6 nm, found in the literature [8–10]. The roughness of the SDS deposit is around 4.8 nm using 2 × 2 μm resolution, and becomes smaller, 3.8 nm, with the 500 × 500 nm resolution.

### 2.3. Infrared Spectroscopy of SDS Adsorbed on Germanium Substrate

**Figure 3:**
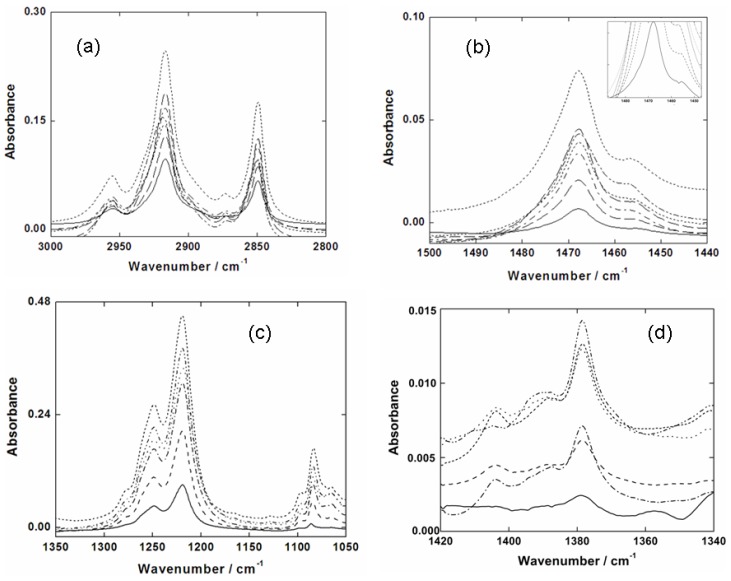
(**a**) The C-H stretching features for SDS molecules deposited on a Ge substrate; (**b**) The CH_2_ scissoring modes (1468 and 1456 cm^−1^); (**c**) The SO_4_^=^ asymmetric (1248 and 1219 cm^−1^) and symmetric (1084 cm^−1^) stretching modes; (**d**) CH^2^ wagging region.

Figure 3a shows the C-H stretching modes for SDS deposited on a Ge substrate. The CH_3_ asymmetric (2955 cm^−1^), the CH_2_ asymmetric (2917 cm^−1^), CH_3_ symmetric (2873 cm^−1^), and CH_2_ symmetric (2850 cm^−1^) stretching bands are shown for different SDS concentrations on a liquid subphase. The CH_3_ asymmetric and symmetric stretching bands are weaker, as expected, than those for the CH_2_ ones. The CH_2_ asymmetric and symmetric stretching bands are the two strongest bands in this region, which may be used to ascribe the packing and conformation of SDS molecules on the Ge substrate [6]. The absorbance ratio between the CH_2_ asymmetric and symmetric stretching features is very constant, 2.4 ± 0.1. The CH_2_ asymmetric stretching mode appears at 2917 cm^−1^, suggesting an ordered hydrocarbon chain in an all-trans CH_2_ conformation [38–40]. Furthermore, a shift was found in the CH_2_ asymmetric stretching band from 2918 to 2920 cm^−1^, which depends on the packing of the SDS molecules.

The CH_2_ scissoring modes of the SDS molecules deposited on the Ge substrate are seen at 1468 cm^−1^ in Figure 3b. This band is very sensitive to chain interactions as well as the packing organization of the methylene chain [41–44]. The band has a shoulder at 1457 cm^−1^, which decreases as the number of SDS molecules deposited on the Ge substrate decreases, becoming a singlet at 1468 cm^−1^. The feature around 1466 cm^−1^ broadens and decreases its intensity, which are both reasonable signs of reduction in chain interactions being accompanied by an increase in chain motion [41–45].

The methylene wagging modes of the hydrocarbon chain of the surfactants are located in the region of 1300–1400 cm^−1^ (see Figure 3d). These bands are known to exhibit peaks with characteristic frequencies for different conformers or high energy rotamers, specifically for structures that contain a gauche orientation [38–40]. In this study, a peak at 1378 cm^−1^ was found which is an indicative of a gauche-trans-gauche (g-t-g) conformation, or an umbrella deformation. This g-t-g conformation may affect the degree of order of the SDS molecules deposited in this study.

In Figure 3c one can see the degenerate SO_4_^=^ asymmetric stretching modes at 1219 and 1249 cm^−1^ of the SDS molecule deposited on the Ge substrate. As the packing of the acyl chains is related to the splitting of the CH_2_ scissoring band, the symmetry of the SO_4_^=^ group similarly produces the SO_4_^=^ asymmetric band splitting as well. In fact, the SO_4_^=^ group is very asymmetric because of the two inequivalent SO_4_^=^ groups that face each other and have a slight dihedral angle between the two pseudo C_3v_ axes. The different oxygen atoms in the SO_4_^=^ group reduce the C_3v_ symmetry to a single mirror plane, resulting in a strongly split band, similar to that found for the CH_2_ scissoring mode [6,35]. Li and Tripp [13] suggested that the decrease of the SO_4_^=^ headgroup symmetry results in changes of lateral electrostatic interactions. A splitting of 30 cm^−1^ was found to occur in our experiments, which is similar to the splitting found by other studies [13,46]. Nevertheless, this value is higher than those reported for SDS crystalline phase [6] and lower than those predicted for bulk deposition [5,7,47,48], liquid crystals [6,49] and for SDS interacting with charged particles [50].

While Sperline [6] has assigned the presence of a medium intensity band at 1061 cm^−1^ as an indicative of micellar SDS, in our study the SO_4_^=^ symmetric stretching band was found at 1084 cm^−1^. It is important to note that this value is shifted to higher wavenumbers as compared to the results in the literature [13,43]. Applying quantum chemical calculation was possible to observe that in the absence of the counterion the frequency shift almost 30 cm^−1^ to higher values, while when the headgroup is hydrated this band shift to lower values, between 10 and 16 cm^−1^ [51]. In addition, two shoulders of the SO_4_^=^ symmetric stretching band were also found at 1097 and 1065 cm^−1^.

Li and Tripp [13] observed a higher intensity in the band at 1249 cm^−1^ than that one at 1219 cm^−1^. On the other hand, the opposite was observed in this study. Also, the difference in intensity between these two bands is larger than that reported by Li and Tripp [13]. This may indicate a lateral interaction of the SO_4_^=^ group with itself or an interaction of SO_4_^=^ with the germanium substrate. Nevertheless, the absorbance increase in the shoulder at 1278 cm^−1^ may also be an indicative of an interaction between the neighboring SO_4_^=^ groups. Therefore, the continuous increasing in the shoulder may be due to the lateral repulsion among headgroups.

Scheuring and Weers [43] observed that the SO_4_^=^ symmetric stretching mode is shifted to higher wavenumbers. The explanation for this shift is the loss of interaction of the SDS headgroup with counterions, suggesting that the shift of the SO_4_^=^ symmetric stretching mode to 1086 cm^−1^ in our investigation is understood by the lack of counterion interaction. Theoretical calculations show that there is a shift of 16–24 cm^−1^ in the absence of the counterion, which is in good agreement with the experimental result presented in this study [51]. The effect of water was also considered on SO_4_^=^ vibrational modes. The shift is predicted to be small. In addition, the shift of the headgroup bands may also be attributed to a possible interaction with the germanium substrate, which is a potential problem with all ATR techniques [52]. Many ATR substrates are highly polar and may perturb the headgroups [52]. However, this problem is not relevant in this situation due to the small interaction between SDS and the Ge substrate.

### 2.4. Linear Dichroism Measurements

Table 1 presents the absorbance of the CH_2_ asymmetric (ν_a_), CH_2_ symmetric (ν_s_), and CH_3_ asymmetric (ν_a_) stretching features for SDS molecules adsorbed on the Ge substrate. The linear dichroic ratios (A_s_/A_p_ = LD = A_⊥_/A_//_), orientation angle (γ), and order parameter (S) calculated from the absorbance of these features are also shown in Table 1. The SO_4_^=^ asymmetric stretching feature has a shoulder and the SO_4_^=^ symmetric is extremely weak. Consequently, it is difficult to attribute any degree of order from these features. With the exception of the SO_4_^=^ symmetric stretching band, the LD ratios are always lower than 1, which indicates that the SDS molecules are slightly perpendicular to the Ge surface. If the SDS molecules are likely to be positioned at an angle γ from the normal to the surface, it is possible to use the uniaxial orientation model to evaluate the degree of order in SDS molecules. It is important to note that the transition dipole moments of the CH_2_ asymmetric and symmetric stretching vibrations are parallel to the hydrocarbon chain axis, while the CH_3_ asymmetric stretching one is perpendicular. Thus, it is used the assumption that α is 90° only for the CH_3_ asymmetric stretching features, while α = 0° is used for the CH_2_ asymmetric and symmetric stretching bands. For an orientation perpendicular to the surface plane, Neivandt *et al.* [53] predicted a linear dichroic ratio value of 1.22 for cetyltrimethylammonium bromide while the work of Haller and Rice [54] showed a range of 1.23–1.28 for a thin film of stearic acid. As can be seen in this study, the LD values for CH_2_ and CH_3_ range from 0.569 to 0.775, which is a good indication for a random orientation. These values were also observed in previous studies [53,55].

The orientation angle, γ, and calculated order parameter, S, are presented in Table 1. From this model, it is straightforward to estimate that the SDS molecules are tilted by 47.6 ± 0.6 degrees from the normal axis, which is in agreement with same angles presented in the literature [30,53,56], which is significantly less than 90° to the surface. In principle, if the order parameter is equal to 1.0, it would mean a perfect order perpendicular to the surface, while a value of −0.5 would indicate a perfect order parallel to the surface. In addition, in the case of S = 0.0 would imply an isotropic distribution or perfect disorder [30,36,37,56]. Nevertheless, the order parameter is calculated to be 0.18 ± 0.01, which indicates that the SDS molecules are not ordered in parallel, and it is most likely that they are being organized with no preferred orientation relative to surface. However, a certain minor alignment may also occur. An important point is that the partition coefficient of SDS molecules in both phases may affect the orientation of the SDS molecules. As is well known, chloroform presents a higher partition coefficient than water. Using water as subphase organizes the hydrocarbon chain pointing it out to the air. On the other hand, apolar solvent leaves the hydrocarbon chain randomly oriented on the air-liquid interface, producing a disordered deposition as reported in this study.

## 3. Experimental Section

Electrophoresis purity grade SDS (purity > 99%) was obtained from Bio-Rad laboratory. Methanol, CH_3_OH, and chloroform, CHCl_3_ (HPLC grade) were purchased from J. T Baker and used as received. SDS/CHCl_3_ solutions at the desired concentration were prepared by using aliquots of a stock solution (0.5 mol·L^−1^) of SDS dissolved in CH_3_OH and the CH_3_OH concentration in the final solution was negligible. A Teflon trough (11 × 10 × 50 mm) was used to accommodate the germanium ATR substrate on the vertical position in order to investigate the deposition dependency on their dimensions. Then, the trough was filled with CHCl_3_ and few microliters of the final SDS/CHCl_3_ solution were dribbled on the CHCl_3_/air interface. The surfactant depositions were obtained by transferring it onto a Ge ATR substrate. CHCl_3_ was eliminated from the trough by slowly pumping it out with a peristaltic pump (P-1 model, Pharmacia) at a speed of 0.1 mL·min^−1^. Then, the Ge substrate with the surfactant deposition was put in a desiccator coupled with a vacuum pump operating at a pressure of ~1 × 10^−2^ Torr to allow the remaining excess solvent to be eliminated, during 10 min. The whole equipment was kept in a clean environment at room temperature to avoid complications with the presence of dust particles. The germanium substrate was used as received, being mainly hydrophilic as it could be visually observed by the formation of the meniscus.

The infrared spectra of transferred SDS molecules were collected in a Varian/Digilab FTS7000 spectrometer equipped with a high sensitivity narrow band liquid-nitrogen-cooled Mercury-Cadmium-Tellurium (MCT) detector. The sampling was performed using a Horizon ATR accessory (Harrick Scientific Inc.). The ATR theory and accessory are fully described in the literature [43]. It consists of a set of two plane mirrors to direct the infrared beam into the ATR germanium element and then to the MCT detector. The ATR germanium element is a single-pass trapezoid with dimension of 2 × 10 × 50 mm and a bevel angle (θ) of 45°. The length (l) and thickness (t) of the ATR element determine the number of reflections (N) by the formula N = l t^−1^ tanθ, which gives 25 internal reflections. For each spectrum, 128 single beam scans were averaged with 1 cm^−1^ resolution for the reference and sample. Prior to deposition, the ATR substrate was cleaned with a suitable procedure [52] until the CH_2_ signal was eliminated (cleaned with chloroform, isopropanol, methanol and water; in this following order). The reference spectrum was obtained by transmitting the infrared beam along the ATR substrate alone, after which the sample spectrum was taken immediately after transferring surfactant molecules onto the ATR element.

The experiments with polarized light were conducted using a holographic infrared polarizer with BaF_2_ substrate (Cambridge Physical Sciences, Model IGP228), which has a useful transmission range from 1000 to 50000 cm^−1^. The electric field components depend on the refractive indices of the Ge ATR element (*n*_1_ = 4.0), sample (*n*_2_ = 1.5), upper phase (*n*_3_ = 1.0), and on the sample thickness. The molecules deposited onto germanium element in these studies are expected to be much thinner than the penetration depth of the evanescent wave. In this case, the electric field can be assumed to be constant within the sample. The electric field components (Equations 1–3) are then given by [52,57,58]:

(1) $$\left[E_{y}^{2}=\frac{4n_{1}^{2}\;{\text{cos}}^{2}\;\theta }{n_{1}^{2}-n_{3}^{2}}\right]$$

(2) $$\left[E_{x}^{2}=E_{y}^{2}\frac{n_{1}^{2}-2n_{3}^{2}}{n_{1}^{2}-n_{3}^{2}}\right]$$

(3) $$\left[E_{z}^{2}=E_{y}^{2}\frac{n_{1}^{2}n_{3}^{2}/n_{2}^{2}}{n_{1}^{2}-n_{3}^{2}}\right]$$

The linear dichroic ratio (LD) (Equation 4) is defined as the ratio of the absorbance for radiation perpendicularly polarized (*A_s_*) to the plane of incidence to that polarized parallel (*A_p_*) to the plane of incidence:

(4) $$\left[LD=\frac{A_{s}}{A_{p}}=\frac{A_{TE}}{A_{TM}}\right]$$

where *A* is the integrated absorbance, *TE* is the transverse electric field or perpendicular field (*E_y_*), and *TM* is the transverse magnetic field or parallel field (= *E_x_* + *E_z_*). For randomly oriented molecules and a given band, the molecular TM vectors are equally distributed about the axes (Equation 5) [56] and

(5) $$\left[LD=\frac{A_{s}}{A_{p}}=\frac{E_{y}^{2}}{E_{x}^{2}+E_{z}^{2}}\right]$$

on the other hand, the uniaxial orientation model [56,58,59] is more appropriate to represent our experiment which has no external force that causes the absorbed surfactant to orient laterally. This model considers two axial symmetric distributions: (1) the transition dipole moment about the chain axis centered at an angle α, and (2) the chain axis about the Z axis centered at an angle γ. In this case, the LD ratio (Equation 6) is given by

(6) $$\left[LD=\frac{A_{s}}{A_{p}}=\frac{E_{y}^{2}(1+{\text{cos}}^{2}\;\gamma )}{E_{x}^{2}(1+{\text{cos}}^{2}\;\gamma )+2E_{z}^{2}\;{\text{sin}}^{2}\;\gamma }\right]$$

using the assumption that α is 90° for the vibration mode of interest. Using the assumption that the transition dipole moment of the vibration mode has an angle of 0° about the chain axis, the LD ratio is represented by (Equation 7):

(7) $$\left[LD=\frac{A_{s}}{A_{p}}=\frac{E_{y}^{2}\;{\text{sin}}^{2}\;\gamma }{E_{x}^{2}\;{\text{sin}}^{2}\;\gamma +2E_{z}^{2}\;{\text{cos}}^{2}\;\gamma }\right]$$

The extent of the orientation can also be described by using a cone model with a fixed opening angle represented by γ, which is related to the order parameter, S (Equation 8) [56–60]:

(8) $$\left[S=1-\frac{3}{2}{\text{sin}}^{2}\;\gamma \right]$$

The smaller this angle is, the better are the molecules aligned. The perfect alignment corresponds to an order parameter of S equal to 1 and an opening angle γ = 0.

The topography was observed by using the AFM technique. AFM experiments were performed with a Nanoscope^®^IIIa Multimode™ of Digital Instruments, and the images were obtained under ambient conditions in the tapping mode. A 250 μm long silicon cantilever (scan rate of 2 Hz) was used with a spring constant of 70 N m^−1^ and two scan areas, 2 × 2 μm and 500 × 500 nm, were monitored. The thickness was also estimated using the AFM system. The organic material was removed from the top to the bottom of the Ge substrate without damaging the substrate surface by using an aged silicon cantilever. Under these conditions, the depth profile of the furrow created upon complete removal provided a good estimate of the local thickness. While the furrow was produced with the slow scanning motion disabled, *i.e.*, the tip was made to run back and forth on the same line, the contrast change in the image display was monitored. The methodology to obtain the furrow was fully described in the literature [61]. Before the SDS deposition on germanium crystal, the roughness of germanium ATR crystal was polished to an average surface roughness of ~2.0 nm. Further AFM analysis of germanium ATR element can be seen in previous literature [62].

## 4. Conclusions

This study describes the SDS deposition on Ge substrate by using Fourier transform infrared-attenuated total reflection (FTIR-ATR) spectroscopy and atomic force microscopy (AFM). The maximum amount deposited is (5.0 ± 0.3) × 10^14^ molecules·cm^−2^ in CHCl_3_, nevertheless, with the use of CCl_4_ as subphase, the SDS deposition is 48% lower than with CHCl_3_. These small values may be due to (i) the weak interaction between SDS and germanium surface and also (ii) to the kind of subphase employed. In general, when it is seen a value of 2917 cm^−1^ for ν_a_ band is a good indication of the organization of SDS molecules. Nevertheless, the AFM images show that SDS molecules are highly disordered, indicating that the assumption applied for *all trans* CH_2_ configuration must be carefully exercised for surfactants in general. In addition, linear dichroism analysis also confirms what is seen by AFM results. The order parameter is ≈2.0, which indicates that the SDS molecules are not paralleling ordered, and it is most likely that they are being organized with no preferred orientation relative to surface; however, a certain minor alignment may also occur. The main reason may be due to the apolar solvent, which leaves surfactant molecules randomly oriented on the air-liquid interface, producing a disordered deposition as reported in this study. Therefore, the results presented here will contribute significantly to future works which use apolar solvents as subphase.

## Figures and Tables

**Table 1:** The absorbances of CH_2_ asymmetric (ν_a_), CH_2_ symmetric (ν_s_), and CH_3_ asymmetric (ν_a_) stretching features at different polarization angles, the linear dichroic ratios (LD), orientation angle (γ), and order parameter (S) for the SDS molecules on the Ge substrate.

	CH_2_		CH_3_

	ν_a_ (α = 0)	ν_s_ (α = 0)	ν_a_ (α = 90)
A_⊥_	1.591	0.724	0.251
A_//_	2.703	1.272	0.324
LD	0.589	0.569	0.775
γ (α = 0°)	48.1	47.0	-
S (α = 0°)	0.169	0.198	-
γ (α = 90°)	-	-	47.7
S (α = 90°)	-	-	0.179
